# Modified Mesoporous Silica (SBA–15) with Trithiane as a new effective adsorbent for mercury ions removal from aqueous environment

**DOI:** 10.1186/2052-336X-12-100

**Published:** 2014-06-25

**Authors:** Mehdi Esmaeili Bidhendi, Gholam Reza Nabi Bidhendi, Nasser Mehrdadi, Hamid Rashedi

**Affiliations:** 1 Department of Environmental Engineering, Graduate Faculty of Environment, University of Tehran, Tehran, Iran; 2 Department of Chemical Engineering, Faculty of Engineering, University of Tehran, Tehran, Iran

**Keywords:** Mercury, Mesoporous Silica, Adsorbent, Modified, Wastewater, SBA–15, Trithiane

## Abstract

**Background:**

Removal of mercury from aqueous environment has been highly regarded in recent years and different methods have been tested for this purpose. One of the most effective ways for mercury ions (Hg^+2^) removal is the use of modified nano porous compounds. Hence, in this work a new physical modification of mesoporous silica (SBA-15) with 1, 3, 5 (Trithiane) as modifier ligand and its application for the removal of Hg^+2^ from aqueous environment has been investigated. SBA-15 and Trithiane were synthesized and the presence of ligand in the silica framework was demonstrated by FTIR spectrum. The amounts of Hg^+2^ in the samples were determined by cold vapor generation high resolution continuum source atomic absorption spectroscopy. Also, the effects of pH, stirring time and weight of modified SBA-15 as three major parameters for effective adsorption of Hg^+2^ were studied.

**Results:**

The important parameter for the modification of the adsorbent was Modification ratio between ligand and adsorbent in solution which was 1.5. The results showed that the best Hg^+2^ removal condition was achieved at pH = 5.0, stirring time 15 min and 15.0 mg of modified adsorbent. Moreover, the maximum percentage removal of Hg^+2^ and the capacity of adsorbent were 85% and 10.6 mg of Hg^+2^/g modified SBA-15, respectively.

**Conclusions:**

To sum up, the present investigation introduced a new modified nano porous compound as an efficient adsorbent for removal of Hg^+2^ from aqueous environment.

## Introduction

Mercury as a kind of heavy metal is present in industrial wastewater such as chemical industry, mining, refining petrochemical and a wide variety of industrial activities [1,2]. This toxic and hazardous chemical compound has harmful effects on the environment and human health. In fact, mercury due to its bioaccumulation property the same as other heavy metals [3], has damaging effects on vital organs such as heart, brain, liver and fatty texture and will result in different cancers. Therefore, this compound is considered as one of the most toxic metals found in the environment and a main source of pollution [4,5]. Hence, nowadays mercury ions removal from aqueous environment (water & wastewater) as one of the major challenges for hygienic management of societies has been the subject of extensive technological research [6]. Several methods including solvent extraction chemical precipitation, vacuum evaporation, membrane technologies, ion exchange, adsorption and membrane separation have been developed and used to remove Hg^+2^ from environment [1,7-10], but some of these methods such as adsorption owing to different problems like low mechanical and thermal stability and a weak chemical union with the Hg^+2^, are often costly or inefficient for removing Hg^+2^ from dilute solutions [1,10,11]. Thus, the scientific community has felt obliged to look for a new method to remove Hg^+2^ from the environment [10]. Hence, synthesis and modification of adsorbents for the removal Hg^+2^ and other heavy metals ions from water and wastewater is a continuing research objective for the control of environmental pollution [12-17]. In this regard, the development of modified mesoporous materials based on MCM-41 and SBA-15 [18] because of their great advantages among large surface area, uniform pore size and controlled surface chemistry for adsorption applications including removal and determination of metal ions [19-24], radio nuclides [25], dyes [26], organics [27], and anionic complexes [28,29], has generated considerable interest [30]. According to the abovementioned, SBA-15 as one of the main types of mesoporous silica materials [31] is a highly common material possessing a regular two dimensional hexagonal array of channels [30]. This material is prepared with non-ionic amphiphilic triblock copolymer micelles as the template under acidic reaction conditions [10]. The pore size of this material typically can be varied between 7–10 nm [30], by varying the synthesis temperature (between 30 and 90°C) and the template [10]. Furthermore, a variety of functional groups such as organic ligands can be grafted or incorporated chemically or physically on the surface of mesoporous channels and prepare highly effective and selective adsorbents [32,33]. Therefore, in this research, 1, 3, 5 Trithiane due to its ability to adsorb Hg^+2^ has been chosen as the ligand for the modification of SBA-15 resulting in a new adsorbent for removing Hg^+2^ from aqueous environment and the Batch method was applied in order to examine the capability of this new adsorbent.

## Materials and methods

### Reagents and chemicals

All chemicals and reagents used in this research were of analytical grade purchased from Merck Company (Darmstadt, Germany). Also, doubly distilled deionized water was used throughout for preparing all solutions. A stock solution (1000 mg L^-1^) of Hg^+2^ was prepared by dissolving the appropriate amounts of Hg (NO_3_)_2_ in doubly distilled deionized water and also a stock standard acetate buffer solution with pH 5.0 was prepared by mixing 14.8 mL 0.2 M acetic acid with 35.2 mL 0.2 M sodium acetate trihydrate and volume with doubly distilled deionized water to 100 mL.

### Apparatus

An Analytik Jena cold vapor generation high resolution continuum source atomic absorption spectroscopy (model: contrAA 700) was used for the determination of Hg^+2^ in the samples. The optimized default conditions of this instrument conformed to Aspect CS 1.5.6 software released by the company. FTIR analyses of the modified and non-modified SBA-15 were performed in a Fourier transform infrared spectroscopy (Bruker-Tensor 27). The samples pH values were measured by a Metrohm pH-meter (model: 713, Herisau, Switzerland) equipped with a glass-combined electrode.

### Synthesis of SBA-15

SBA-15 preparation was based on the procedure in the literature [34]. 2.0 g of triblock copolymer P123 (EO_20_PO_70_EO_20_) was dissolved in 60.0 g of 2 M Hydrochloric acid (HCl) aqueous solution stirred at 40°C. Then 4.3 g of Tetraethylorthosilicate (TEOS) was added to the homogeneous solution and stirred at this temperature for 24 h. Finally, it was heated to 100°C and held at this temperature for 24 h under static conditions. The prepared sample was filtered, and then washed with water and air-dried at room temperature. The removal of the template was carried out at 550°C in air for 5 h.

### Synthesis of 1, 3, 5 Trithiane as Modifier

**Figure 1:**
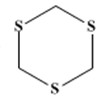
Chemical structure of Trithiane.

Since SBA-15 as the base adsorbent is not efficient for metal ions removal from aqueous environment, it needs modifying. One organic ligand that has the ability to form complex with Hg^+2^ is 1, 3, 5 Trithiane which was previously reported by research in literature [35]. This organic compound (Figure 1) as modifier ligand was prepared based on the procedure in literature [36]. A mixture of 32.6 g of Formaldehyde solution %36 (w/w) and 70 cc of concentrated Hydrochloric acid were used and then Hydrogen sulfide passed through the solution until no more precipitate was formed. In order to facilitate the process, the accumulated mass of crystals was removed by filtration from time to time. Afterward, the product was purified by the inverted filtration method.

### Modification of SBA -15

Modification of the prepared SBA-15 was performed by dissolving 60 mg of Trithiane in adequate amount of Acetonitrile and adding 40 mg of SBA-15 and mixing for 15 min. Next, the solution was filtered and the solid phase as the functionalized product was exposed to atmosphere and used as a new sorbent for this research.

### Metal adsorption experiments

The general procedure for removal of Hg^+2^ by the modified SBA-15 was as follows: A batch system was employed for the removal of Hg^+2^ and removing was performed in a beaker containing 10 mL (10 mg L^-1^) Hg^+2^ solutions. About 10 mg modified SBA-15 was added to the solution. Then the mixture was stirred for 10 min to remove Hg^+2^ from the solution. Finally, filtration was performed and Hg^+2^ concentrations were determined by cold vapor generation high resolution continuum source atomic absorption spectroscopy (model: contrAA 700).

## Results and discussion

SBA-15 modification seems essential through suitable ligand because this mesoporous adsorbent has a major problem removing Hg^+2^ from aqueous environment selectively and efficiently. Previous studies [35] indicated that 1, 3, 5 Trithiane as organic ligand has an efficient interaction with Hg^+2^. Hence, it was used in the modification of SBA-15 for the removal of Hg^+2^. The preliminary experiments showed that the removal of Hg^+2^ ions by non-modified and modified SBA-15 was obviously different and the former cannot remove Hg^+2^ quantitatively.

### FTIR analysis

**Figure 2:**
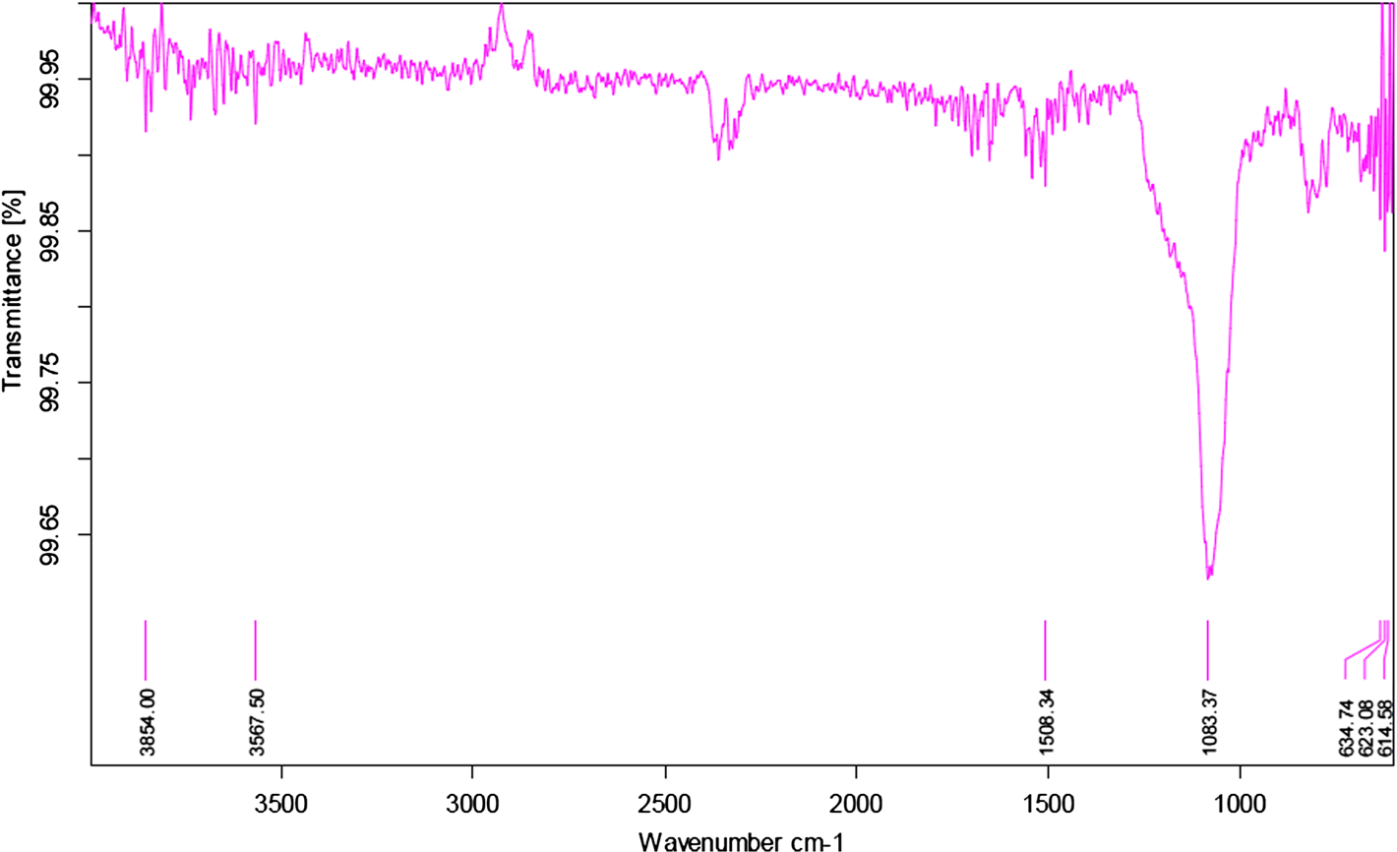
FTIR spectrum of modified SBA-15.

For the characterization of the modified SBA-15, infrared spectrum was recorded on Bruker-Tensor 27 spectrophotometer in the region 400 – 4000 cm^-1^ using spectra quality KBr powder. According to Figure 2 the distinguished band at 1508 cm^-1^ can be assigned to Trithiane.

### Effect of Modified Ratio (Ligand / SBA)

Modification ratio between ligand and adsorbent shown in this research as (Ligand / SBA-15) is one of the parameters affecting the prepared efficient modified adsorbent. In this study, mesoporous silica has been modified by Trithiane ligand by 3 modification ratios as 0.5, 1 and 1.5 (w/w). Then all the modified adsorbents were tested for removing Hg^+2^ from the samples. The results showed that the adsorbent modified by 1.5 (Ligand/SBA-15 w/w) has the maximum percentage of Hg^+2^ removal from the samples compared to the other two modification ratios. According to the obtained results, higher modification ratios were not studied in this research because the modified adsorbent has to be economical. On the other hand, the Hg^+2^ removal process revealed that by raising the modification ratio from 0.5 to 1 and 1 to 1.5, the percentage of removal increased slightly. Therefore, in the subsequent experiments we used 1.5 (Ligand / SBA-15 w/w) modification ratio.

### Effect of pH

**Figure 3:**
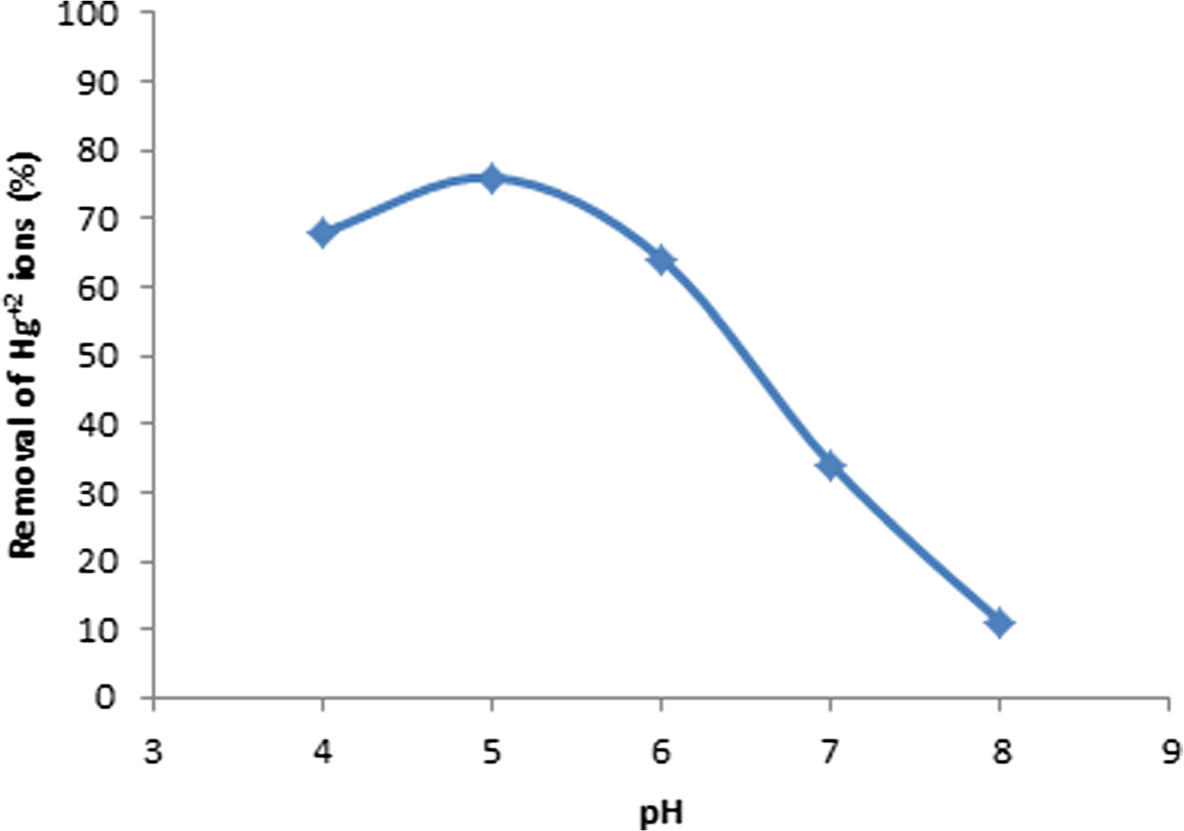
**Effect of pH on the removal of Hg**^**+2**^**.**

The aqueous samples pH adjustment is one of the major parameters in order to obtain efficient removal of toxic metal ions by specific adsorbents such as modified mesoporous silica. Hence, in this research the effect of pH on the removal of Hg^+2^ ions with modified adsorbent was studied at different pH values ranging from 4.0 to 8.0. As shown in Figure 3, the percentage of Hg^+2^ ions adsorption increased significantly between pH 4.0 – 5.0 and then at pH higher than 5.0 decreased extremely. In pH values higher than 8.0, modified mesoporous silica may be hydrolyzed in alkali solutions due to the breaking of the Si-O-Si bonds by hydroxide ions attack so this range of pH was not studied. Also, pH values lower than 4.0, were not studied in this research because this range due to decreasing pH value causes the ligand to be surrounded by H^+^ ions and prevents Hg^+2^ from reaching the ligand to form complexes for its removal from the environment. Therefore, pH value of the sample solutions was adjusted at 5.0 in the subsequent experiments.

### Effect of Weight of Adsorbent

**Figure 4:**
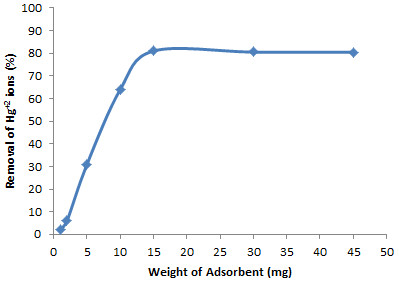
**Effect of amount of adsorbent on the removal of Hg**^**+2**^**.**

The optimum amount of the modified SBA-15 for maximum removal of Hg^+2^ was investigated by testing different amounts of modified sorbent (1, 2, 5, 10, 15, 30 and 45 mg) for Hg^+2^ uptake from the samples. As can be seen from Figure 4 in which the results are given, by raising the sorbent amount from 1 to 15 mg the percentage of Hg^+2^ removal increased significantly and in the range of 15 to 45 mg the percentage of removal was almost constant. Hence, in the experiments 15.0 mg of modified SBA-15 was used thereafter.

### Effect of the stirring time on removal yield

**Figure 5:**
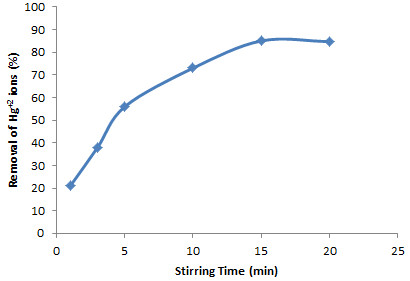
**Effect of amount of stirring time on the removal of Hg**^**+2**^**.**

The effect of stirring time on Hg^+2^ ions removal was investigated by performing a series of removal experiments with the same conditions and at several times (1, 3, 5, 10, 15 and 20 min). According to the results shown in Figure 5, Hg^+2^ removal up to 15 min increased and then was nearly constant. Therefore, the 15-min stirring time was used afterward.

### Capacity of the modified SBA-15

The adsorbent capacity is an important parameter in adsorption processes because it determines how much adsorbent is required to quantitatively remove a specific amount of metal ions from the solutions [37]. Thus, the adsorption capacity of Hg^+2^ per unit weight of the modified adsorbent at time **t**, **q**_**t**_ (mg g^-1^), was calculated from the mass balance:

$$q_{t}=\frac{\left(C_{0}-C_{t}\right)\times V}{m}$$

Where **C**_**0**_ (mg L^-1^) is the initial concentration of Hg^+2^ and **C**_**t**_ (mg L^-1^) is Hg^+2^ concentration at time t. **V** is the volume of Hg^+2^ solutions and **m** is the mass of the modified mesoporous. The capacity of the modified adsorbent was found to be 10.6 mg of Hg^+2^/g modified SBA-15.

## Conclusion

In the present research, new and effective modified mesoporous silica was prepared and tested in batch mode for Hg^+2^ removal from the aqueous samples. The results demonstrated a successful application of modified SBA-15 for the effective removal of Hg^+2^ as a rapid and easy method. Maximum percentage of removal happened at pH equal to 5.0, 15-min stirring time and using 15 mg of modified adsorbent. As adsorbents are used in wastewater treatment plants, the preparation of an applicable form of adsorbent such as an adsorption column and the determination of its effective parameters proves necessary so the authors will focus on this target in the future.

## Competing interests

The authors declare that they have no competing interests.

## Authors’ contributions

This research is a part of the thesis by MEB who prepared the literature survey and performed the experiments. NM and HR participated in the design of the study, data analysis, and manuscript preparation. GRNB was the advisor. All the authors have read and approved the final manuscript.

## References

[B1] Rostamian R Najafi M Rafati AA Synthesis and characterization of thiol-functionalized silica nano hollow sphere as a novel adsorbent for removal of poisonous heavy metal ions from water: Kinetics, isotherms and error analysis Chem Eng J 2011 171 1004 1011 10.1016/j.cej.2011.04.051

[B2] Aguado J Arsuaga JM Arencibia A Lindo M Gascon V Aqueous heavy metals removal by adsorption on amine-functionalized mesoporous silica J Hazard Mater 2009 163 213 221 10.1016/j.jhazmat.2008.06.080 18675509

[B3] Boening DW Ecological effects, transport, and fate of mercury: a general review Chemosphere 2000 40 12 1335 1351 10.1016/S0045-6535(99)00283-0 10789973

[B4] Evangelista SM Deoliveira E Castro GR Zara LF Prado AGS Hexagonal mesoporous silica modified with 2-mercaptothiazoline for removing mercury from water solution Surf Sci 2007 601 2194 2202 10.1016/j.susc.2007.03.020

[B5] Esmaeili Bidhendi M Karbassi AR Baghvand A Saeedi M Pejman AH Potential of natural bed soil in adsorption of heavy metals in industrial waste landfill Int J Environ Sci Tech 2010 7 3 545 552 10.1007/BF03326163

[B6] Lee B Kim Y Lee H Yi J Synthesis of functionalized porous via templating method as heavy metal ion adsorbents: the introduction of surface hydrophilicity onto the surface of adsorbents Micropor Mesopor Mater 2001 50 77 90 10.1016/S1387-1811(01)00437-1

[B7] Nabi SA Shahadat M Bushra R Shalla AH Ahmed F Development of composite ion-exchange adsorbent for pollutants removal from environmental wastes Chem Eng J 2010 165 405 412 10.1016/j.cej.2010.08.068

[B8] Najafi M Rostamian R Rafati AA Chemically modified silica gel with thiol group as an adsorbent for retention of some toxic soft metal ions from water and industrial effluent Chem Eng J 2011 168 426 432 10.1016/j.cej.2010.12.064

[B9] Huuha TS Kurniawan TA Sillanpaa MET Removal of silicon from pulping whitewater using integrated treatment of chemical precipitation and evaporation Chem Eng J 2010 158 584 592 10.1016/j.cej.2010.01.058

[B10] Quintanilla DP Hierro ID Fajardo M Sierra I 2-Mercaptothiazoline modified mesoporous silica for mercury removal from aqueous media J Hazard Mater 2006 B134 245 256 10.1016/j.jhazmat.2005.11.004 16326000

[B11] Babel S Kurniawan TA Low-cost adsorbents for heavy metals uptake from contaminated water: a review J Hazard Mater 2003 97 219 243 10.1016/S0304-3894(02)00263-7 12573840

[B12] Mahmoud ME Osman MM Hafez OF Hegazi AH Elmelegy E Removal and preconcentration of lead (II) and other heavy metals from water by alumina adsorbents developed by surface-adsorbed-dithizone Desalination 2010 251 123 130 10.1016/j.desal.2009.08.008

[B13] Mahmoud ME Hafez OF Alrefaay A Osman MM Performance evaluation of hybrid inorganic/organic adsorbents in removal and preconcentration of heavy metals from drinking and industrial waste water Desalination 2010 253 9 15 10.1016/j.desal.2009.11.044

[B14] Mahmoud ME Kenawy IMM Hafez MAH Lashein RR Removal, preconcentration and determination of trace heavy metal ions in water samples by AAS via chemically modified silica gel N-(1-carboxy-6-hydroxy) benzylidenepropylamine ion exchanger Desalination 2010 250 62 70 10.1016/j.desal.2009.09.009

[B15] Burham N Separation and pre concentration system for lead and cadmium determination in natural samples using 2-aminoacetylthiophenol modified polyurethane foam Desalination 2009 249 1199 1205 10.1016/j.desal.2009.04.009

[B16] Ciftci H Yalcin H Eren E Olcucu A Sekerci M Enrichment and determination of Ni^2+^ ions in water samples with a diamino-4-(4-nitro-phenylazo)-1H-pyrazole (PDANP) by using FAAS Desalination 2010 256 48 53 10.1016/j.desal.2010.02.018

[B17] Chakravarty P Sen Sarma N Sarma HP Removal of lead (II) from aqueous solution using heartwood of Areca catechu powder Desalination 2010 256 16 21 10.1016/j.desal.2010.02.029

[B18] Lei B Li B Zhang HR Lu SZ Zheng ZH Li WL Wang Y Mesostructured Silica Chemically Doped with RuII as a Superior Optical Oxygen Sensor Adv Funct Mater 2006 16 14 1883 1891 10.1002/adfm.200500737

[B19] Perez-Quintanilla D Sanchez A Del Hierro I Fajardo M Sierra I Preparation of 2-mercaptobenzenothiazole-derivatived mesoporous silica and of Hg (II) from aqueous solution. J Environ Monit 2006 8 214 222 10.1039/b507983g 16395482

[B20] Perez-Quintanilla D Sanchez A Del Hierro I Fajardo M Sierra I Preconcentration of Zn (II) in water samples using a new hybrid SBA-15-based material J Hazard Mater 2009 166 1449 1458 10.1016/j.jhazmat.2008.12.065 19157702

[B21] Perez-Quintanilla D Sanchez A Del Hierro I Fajardo M Sierra I Solid phase extraction of Pb(II) in water samples using a new hybrid inorganic–organic mesoporous silica prior to its determination by FAAS Microchim Acta 2009 165 291 298 10.1007/s00604-008-0132-0

[B22] Kang T Park Y Choi K Sang Leec J Yi J Ordered mesoporous silica (SBA-15) derivatized with imidazole-containing functionalities as a selective adsorbent of precious metal ions J Mater Chem 2004 14 1043 1049 10.1039/b315829b

[B23] Ganjali MR Hajiaghababaei L Badiei AR Saberyan K Salavati-Niasari M Ziarani GM Behbahani SMR A novel method for fast enrichment and monitoring of hexavalent and trivalent chromium at the PPT level with modified silica MCM-41 and its determination by inductively coupled plasm optical emission spectrometry Quim Nova 2006 29 440 443 10.1590/S0100-40422006000300007

[B24] Ganjali MR Hajiaghababaei L Badiei AR Ziarani GM Tarlani A Novel method for the fast preconcentration and monitoring of a ppt level of lead and copper with a modified hexagonal mesoporous silica compound and inductively coupled plasma atomic emission spectrometry Anal Sci 2004 20 725 729 10.2116/analsci.20.725 15116976

[B25] Ju YH Webb OF Dai S Lin JS Barnes CE Synthesis and characterization of ordered mesoporous anion-exchange inorganic hybrid resins for radionuclide separation Ind Eng Chem Res 2000 39 550 553 10.1021/ie990597v

[B26] Ho KY Mckay G Yeung KL Selective adsorbents from ordered mesoporous silica Langmuir 2003 19 3019 3024 10.1021/la0267084

[B27] Lim MH Stein A Comparative studies of grafting and direct syntheses of inorganic–organic hybrid mesoporous materials Chem Mater 1999 11 3285 3295 10.1021/cm990369r

[B28] Lee B Bao LL Im HJ Dai S Hagaman EW Lin JS Synthesis and characterization of organic–inorganic hybrid mesoporous anion-exchange resin for perrhenate (ReO4 -) anion adsorption Langmuir 2003 19 4246 4252 10.1021/la026960b

[B29] Fryxell GE Liu J Hauser TA Nie Z Ferris KF Mattigod S Gong M Hallen RT Design and synthesis of selective mesoporous anion traps Chem Mater 1999 11 2148 2154 10.1021/cm990104c

[B30] Hajiaghababaei L Badiei A Ganjali MR Heydari S Khaniani Y Mohammadi Ziarani G Highly efficient removal and preconcentration of lead and cadmium cations from water and wastewater samples using ethylenediamine functionalized SBA-15 Desalination 2011 266 182 187 10.1016/j.desal.2010.08.024

[B31] Zhao D Huo Q Feng J Chmelka BF Stucky GD Nonionic triblock and star diblock copolymer and ologomeric surfactant syntheses of highly ordered, hydrothermally stable, mesoporous silica structures J Am Chem Soc 1998 120 6024 6037 10.1021/ja974025i

[B32] Badiei A Norouzi P Tousi F Study of electrochemical behavior and adsorption mechanism of [Co(en)_2_C_l2_] + on mesoporous modified carbon paste electrode Eur J Sci Res 2005 12 39 45

[B33] Badiei A Bonneviot L Crowther N Mohammadi Ziarani G Surface tailoring control in micelle templated silica J Organomet Chem 2006 691 5923 5931

[B34] Murty DSR Chakrapani G Preconcentration of rare earth elements on activated carbon and its application to groundwater and sea-water analysis J Anal At Spectrom 1996 11 815 820 10.1039/ja9961100815

[B35] Ercan Ö Aydınb A Removal of Mercury, Antimony, Cadmium and Lead from Aqueous Solution using 1,3,5-Trithiane as an Adsorbent J Braz Chem Soc 2013 24 5 865 872

[B36] Organic Syntheses Coll. Vol. 2, p.610 (1943); Vol. 16, p.81 (1936) [http://www.orgsyn.org/Content/pdfs/procedures/CV2P0610.pdf]

[B37] Dev K Rao GN Preparation and analytical properties of a chelating resin containing bicine groups Talanta 1995 42 591 596 10.1016/0039-9140(95)01452-H 18966268

